# Assessment of Troponin I Levels as a Predictor of Mortality in Acute Decompensated Heart Failure

**DOI:** 10.7759/cureus.48760

**Published:** 2023-11-13

**Authors:** Aravind Sreekumar, Ankit K Sahu, Praveen Aggarwal, Jamshed Nayer, Rajib Narang

**Affiliations:** 1 Emergency Medicine, All India Institute of Medical Sciences, New Delhi, New Delhi, IND; 2 Cardiology, All India Institute of Medical Sciences, New Delhi, New Delhi, IND

**Keywords:** cardiac biomarkers, emergency department, predictors, mortality, troponin i, acute decompensated heart failure

## Abstract

Background: Acute decompensated heart failure (ADHF) is a highly prevalent diagnosis in the emergency department and is associated with high morbidity and mortality. As mortality remains high even in patients discharged from the emergency, it becomes mandatory to identify markers predictive of mortality in order to guide the disposition of such patients. No literature is available on the prognostic significance of Troponin I in ADHF patients in an emergency setting from an Indian standpoint.

Objectives: This study was aimed at identifying the ability of Troponin I levels at presentation to predict one-month mortality in patients with ADHF.

Methods: The study was conducted in the emergency department of a tertiary healthcare center in north India. Serum cardiac Troponin I (cTnI) levels at presentation were assayed in 101 patients and a one-month follow-up was done.

Results: cTnI levels were > 0.02 ng/mL in 51 patients (50.5%). ROC analysis showed an accuracy of 63% in predicting mortality (p < 0.05). Univariate and multivariate analysis showed an OR of 2.58 and 2.74, respectively (p - 0.037 and 0.047, respectively), suggesting cTnI to be a significant predictor of mortality in ADHF. N-terminal proBNP (NT-proBNP) (OR - 2.09; p - 0.229) and left ventricular ejection fraction (OR - 2.01; p - 0.157) were not found to be significant predictors of mortality on regression analysis.

Conclusion: cTnI levels at presentation are a significant predictor of short-term mortality in ADHF and can be used in an emergency setting to guide treatment, disposition, and follow-up plans of these patients.

## Introduction

Heart failure (HF) is defined as symptoms or signs due to abnormalities of cardiac structure or function, with the diagnosis supported by increased natriuretic peptide levels as well as evidence of systemic or pulmonary congestion [1]. Acute decompensated HF (ADHF) is defined as any new-onset or worsening signs and symptoms of HF, requiring urgent hospitalization [2]. The incidence of hospitalizations for ADHF is estimated to be around 1.8 million per annum in India, with an estimated average two-year expenditure of 1.3 lakh INR per patient, signifying the enormous burden of this condition on the healthcare system as well as the patient population [3]. Studies show that while up to a third of patients presenting with ADHF are discharged from the emergency, their short-term mortality remains as high, if not higher than the patients admitted [4]. This mandates the use of prognostic markers in an emergency setting to identify the patients at high risk of short-term mortality in order to modify treatment and disposition plans accordingly. This becomes especially relevant in a developing country like India where large sections of the population still do not have access to quality healthcare systems, and patients are commonly lost to follow-up following presentations to the emergency.

Different markers have been postulated to show an association with poor prognosis in patients with HF. Missov et al. provided the first evidence of cardiac troponin-I (cTn-I) as a potentially sensitive and specific marker of myocardial injury in HF [5]. The high sensitivity and specificity of cTn-I combined with the relative ease of availability of its assay would make it a valuable resource in assessing the severity of the disease and prognostication in patients with ADHF. Although quite a few studies [6-8] have been conducted showing cTn-I as a prognostic marker in ADHF, there has been only one study in the Indian population, with a limited sample size [9]. The present study was done to assess the association between cTn-I levels and one-month mortality in patients presenting to the emergency department (ED) of a tertiary care center in India with ADHF.

## Materials and methods

This was a single-center, prospective, observational study done in the ED of a tertiary care teaching hospital in north India. The study was conducted from August 2020 to June 2022, after obtaining ethical approval from the Institute Ethics Committee (IECPG-290/22.07.2020).

Adult patients (age > 18 years) presenting to the ED with ADHF, were recruited in the study. ADHF was defined as any sudden onset or sudden worsening of symptoms of heart failure (HF) within seven days, HF being defined by the Framingham criteria [10]. Patients with valvular heart disease, congenital heart disease (CHD), acute coronary syndrome (ACS), or myocarditis at the time of presentation to ED were excluded from the study. Patients were recruited after obtaining informed consent from the patient or his/her legally authorized representative. Based on the study by Horwich et al. [7], taking α as 0.05 and β to be 0.1, the sample size for the said study was calculated to be 82. Accounting for possible dropouts, a sample size of 100 was decided.

Data were collected in a predesigned questionnaire. Data collected included demographic profile, presenting symptoms of the patient, previous medical/surgical history, and physical examination findings. Five milliliter blood sample was drawn from the patient at presentation and collected in standard dipotassium edetate vials for Troponin I and NT-proBNP assay. Estimation was done using Radiometer AQT90 Flex Analyzer (Radiometer Medical Aps, Brønshøj, Denmark). Reference ranges for Troponin I and NT-proBNP were <0.02 ng/mL and 70-133 pg/mL, respectively. Bedside echocardiography using Fujifilm Sonosite Ultrasound machine (FUJIFILM Sonosite, WA, USA), was done to determine Left ventricular ejection fraction (EF). The investigator was well trained in the EF assessment in ED prior to the start of the recruitment of study participants. Other investigations included electrocardiograms, chest radiography, serum electrolytes, and serum creatinine. The endpoint of the study was one-month mortality, and this information was collected by contacting the patient or his/her family members by phone one month after the presentation.

Quantitative variables were presented as mean with standard deviation (SD) (if distributed normally) and median with interquartile range (IQR) (if not distributed normally). Qualitative variables were expressed as counts and percentages. The receiver operating characteristics (ROC) curve was used to assess the accuracy of cTn-I, NT-proBNP, and EF values in predicting mortality, and to determine valid cut-offs by the Youden’s-J test. Logistic regression (LR) was used to analyze the predictors of one-month mortality where covariates included demographic parameters, vitals at clinical presentation, cTn-I, NT-proBNP, and EF. The cTn-I, NT-proBNP, and EF values were converted to categorical values using the cut-offs from ROC analysis and entered into the LR model. Only the covariates that were significant predictors of one-month mortality in the univariate analysis were considered for the multivariate LR model. Statistical significance was set at a two-tailed p-value of less than 0.05. The data were collected in Microsoft Excel (Microsoft Corporation, 2018) and all statistical analyses were done with IBM SPSS Statistics for Windows (version 25.0, Released 2020. IBM Corp., Armonk, NY).

## Results

**Figure 1:**
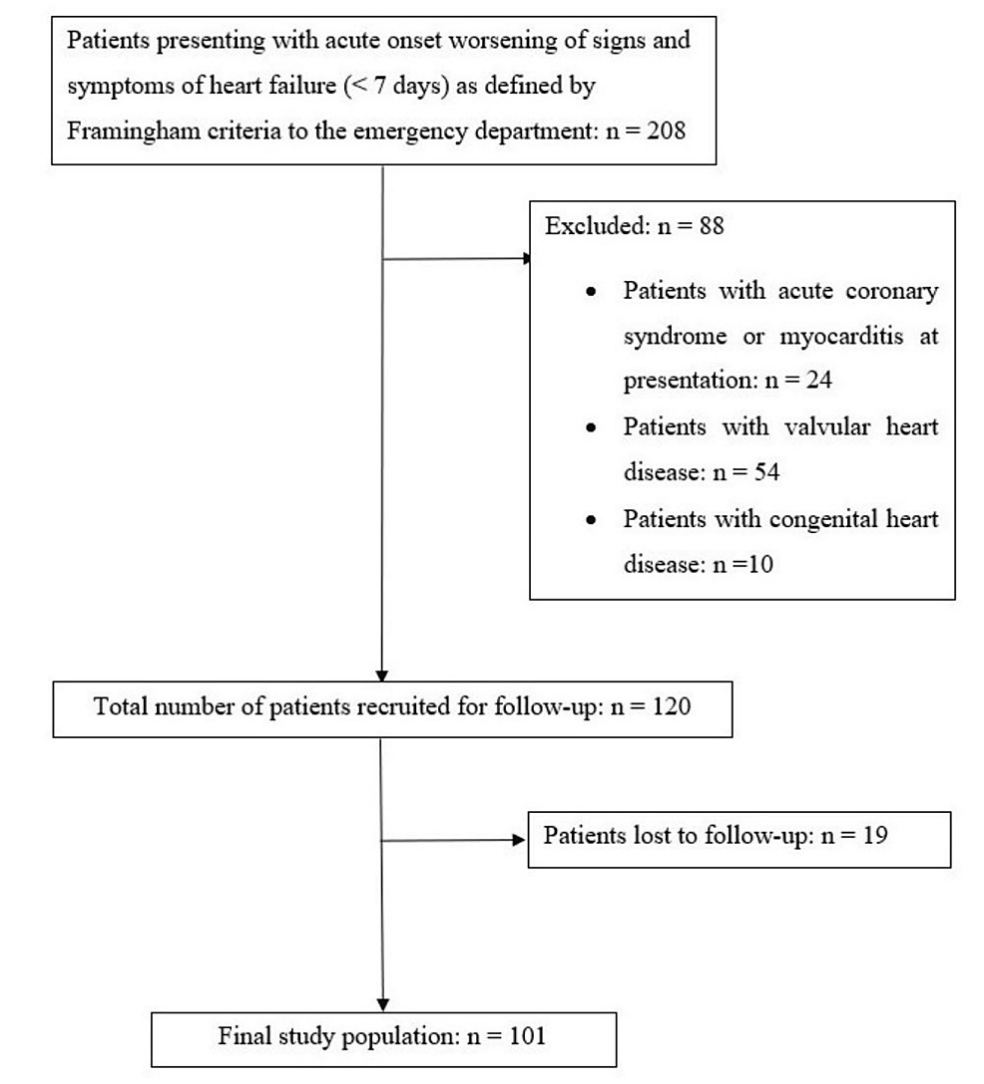
Recruitment of study population

A total of 208 patients presenting with ADHF were screened for the study. Eighty-eight patients were excluded: 24 patients had ACS or myocarditis at presentation, 54 had underlying valvular heart disease and 10 had CHD. Nineteen out of the 120 patients could not be followed up mostly due to logistical reasons owing to the COVID-19 pandemic, resulting in a final sample size of 101 patients for analysis (Figure 1).

The mean age of the patients recruited was 52.4 ±17.3 years with 57 out of 101 patients (56.4%) being male. The most common presenting symptom was shortness of breath (97%) followed by swelling of feet (37.6%) and cough with expectoration (30.7%). Most common comorbid conditions in the studied population were hypertension (50.5%) and diabetes mellitus (35.6%). The detailed demographic characteristics, vitals at presentation and laboratory parameters of the study population are presented in Table 1.

**Table 1:** Patient demographic characteristics, vitals and laboratory parameters at presentation to the emergency department (n = 101)

Parameters	Value – n (%) or mean, SD
Age (mean, SD)	52.4, 17.3
Male gender	57 (56.4%)
Presenting characteristics	
Shortness of breath	98 (97.0%)
Swelling of feet	38 (37.6%)
Cough with expectoration	31 (30.7%)
Decreased urine output	16 (15.8%)
Fever	15 (14.9%)
Abdominal pain	12 (11.9%)
Fatigue	11 (10.9%)
Comorbidities	
Hypertension	51 (50.5%)
Diabetes mellitus	36 (35.6%)
Chronic kidney disease	33 (32.6%)
Coronary artery disease	25 (24.7%)
Obstructive airway disease	19 (18.8%)
No previous comorbidities	16 (15.8%)
Vitals at presentation	
Pulse rate (per minute)	105.4, 25.1
Respiratory rate (per minute)	27.1, 6.011
Systolic blood pressure (in mmHg)	146.9, 42.7
Peripheral oxygen saturation (%)	87, 13
Laboratory values at presentation	
Sodium (mEq/L)	133.1, 14.3
Potassium (mEq/L)	4.9, 1
Creatinine (mg/dL)	3.7, 0.5
Lactate (mmol/L)	2.8, 0.8
Cardiac troponin – I (ng/mL)	0.195, 0.50
NT-proBNP (pg/mL)	16822.1, 12954.6
Ejection fraction (%)	35, 13

The mean values of cTn-I, NT-proBNP and EF were 0.195 ± 0.50 ng/mL, 16822.1 ± 12954.6 pg/mL and 35 ± 13%, respectively. Mean cTnI values were significantly higher in patients who died as compared to those who were alive (0.335 ng/ml vs 0.107 ng/mL, p - 0.007) at one-month follow-up. Similarly, the mean NT-proBNP was significantly higher in the former group as compared to the latter (20,967 pg/mL vs 15,129 pg/mL, p - 0.040). The mean EF tended to be lesser in patients who died at one month, but the difference was not statistically significant (31% vs 37%, p - 0.066). Detailed comparison is depicted in Table 2.

**Table 2:** Comparison of quantitative markers with mortality* *Values expressed as mean (95% confidence interval)

Parameter	Alive	Not Alive	P-value
Cardiac troponin – I (ng/mL)	0.107 (0.023 - 0.191)	0.335 (0.097 – 0.574)	0.007
NT-proBNP (pg/mL)	15129 (12072 – 18185)	20967 (16286 – 25647)	0.040
Ejection fraction (%)	36.5 (33.6 – 39.3)	30.8 (24.9 – 36.7)	0.066

**Figure 2:**
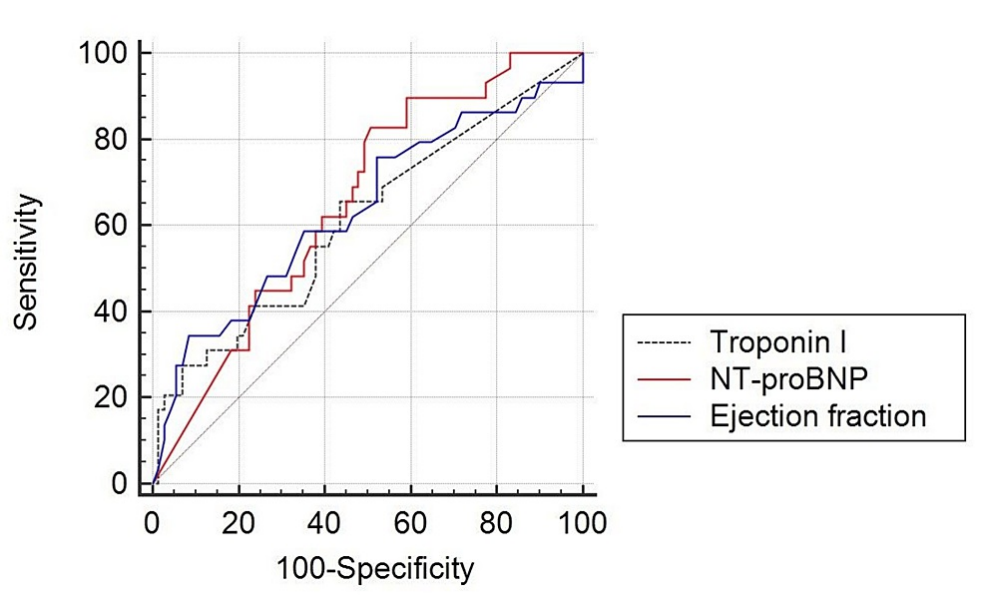
The receiver operating characteristic curve of troponin - I, NT-proBNP and ejection fraction predicting one-month mortality

The ROC curve was prepared to assess the ability of the individual markers to predict mortality and shown in Figure 2.

The AUROC of cTn-I for predicting one-month mortality was 0.625 (95% CI: 0.501-0.749, p - 0.048). A cTn-I value of more than 0.020 ng/mL (cut-off decided by Youden’s-J test) was found to be 66.7% sensitive and 56.3% specific for prediction of mortality. NT-proBNP showed an AUROC of 0.654 (95%CI: 0.543-0.766, p - 0.016). An NT-proBNP cut-off of 10,000 pg/mL showed a sensitivity of 70% and a specificity of 52.11% for prediction of mortality. ROC analysis of EF showed an AUROC of 0.627 (95%CI: 0.501-0.752, p - 0.045). A cut-off of 40% was shown to have a sensitivity of 76.66% and a specificity of 38.28% for predicting one-month mortality. None of the markers were significantly superior to the others in predicting mortality as per ROC analysis (p > 0.05). The diagnostic statistics of all parameters in predicting study outcome are reported in Table 3.

**Table 3:** Diagnostic statistics of markers under evaluation in predicting one-month mortality

Parameter	Sensitivity	Specificity	Negative predictive value	Positive predictive value
CTn-I (more than 0.020 ng/mL)	66.67%	56.3%	80%	39.22
NT-proBNP (more than 10000 pg/mL)	70%	52.11%	80.43%	38.18%
Ejection fraction (< 40%)	76.66%	38.28%	79.41%	34.32%

On logistic regression, the covariates that showed a significant association for with mortality were cTn-I and NT-proBNP. A cTn-I value of more than 0.020 ng/mL was found to have an odds ratio (OR) of 2.58 (95%CI: 1.06 - 6.29, p - 0.037) in predicting mortality. An NT-proBNP value of more than 10,000 pg/mL predicted mortality with an OR of 2.54 (95%CI: 1.02 - 6.30, p - 0.045). However, the EF was not able to predict the mortality significantly (OR - 2.02 95%CI: 0.76 - 5.33, p - 0.157). Subsequent multivariate analysis showed only cTn-I to be a significant predictor of mortality (adjusted OR - 2.74, 95% CI: 1.01 -7.43, p - 0.047). The results of univariate and multivariate logistic regression analysis are shown in Table 4.

**Table 4:** The logistic regression analysis for predictors of one-month mortality in acute heart failure patients

	Univariate			Multivariate		
	Odds ratio	95% CI	P-value	Adjusted odds ratio	95% CI	P-value
cTn-I > 0.020 ng/mL	2.581	1.057-6.299	0.037	2.742	1.012-7.434	0.047
NT-proBNP> 10,000 pg/mL	2.539	1.023-6.303	0.045	2.095	0.628-6.983	0.229
Ejection fraction <= 40%	2.016	0.763-5.331	0.157			
Age	1.006	0.981-1.031	0.641			
Female sex	0.570	0.241-1.348	0.200			
Shortness of breath	0.200	0.017-2.295	0.196			
Decreased urine output	0.496	0.130-1.885	0.303			
Respiratory rate > 20/min	1.193	0.494-2.877	0.695			
Systolic blood pressure < 90	0.287	0.060-1.370	0.117			
SpO2 < 94%	0.706	0.293-1.697	0.436			
Lactate > 2.0 mmol/L	1.174	0.495-2.789	0.716			

## Discussion

The mechanisms describing the elevation of cardiac CTn-I in patients of HF are still open to debate. The commonly postulated mechanisms that occur in HF include sub-endocardial ischemia resulting in necrosis of the myocytes [11,12], increased membrane permeability of injured myocytes [13], integrin-mediated release of Troponin by myocytes [14] and alterations in calcium handling activating intracellular protein enzymes [15].

We conducted this prospective observational study to investigate the association of different markers like cTn-I with one-month mortality in ADHF patients in Indian settings. Our study population showed similarities in demographic characteristics to another Indian HF registry [16]. In our study, it was found that the cTn-I, NT-proBNP, and EF at presentation were associated with the study outcome. After adjusting for all variables, only cTn-I was shown to predict mortality significantly.

The cTn-I was elevated (> 0.02 ng/mL) in 50.5% of patients recruited in our study. The ROC analysis showed that cTn-I was 63% accurate in predicting mortality. Arenja et al. found that the cTn-I was raised in 41% of 357 AHF patients (cutoff of 0.028 ng/mL) and cTn-I was 74% accurate in predicting mortality. This discrepancy, however, could be explained by the fact that, unlike our study, this study included patients with ACS. The OR and adjusted OR of raised cTn-I (> 0.02 ng/mL) in predicting mortality were 2.58 and 2.74 in our study, respectively, which were in line with a similar study by Peacock et al. based on the ADHERE database [17]. They concluded that a raised cTn-I (> 0.1 ng/mL) predicted mortality with an adjusted OR of 2.55. Similarly, Horwich et al. studied 238 ADHF patients and found a significant correlation between cTn-I and mortality with a relative risk of 2.1 [7]. You et al. conducted the EFFECT study in Canada, wherein they concluded that cTnI levels > 0.5 ng/mL represented an independent predictor of mortality (adjusted hazard ratio: 1.49) [8].

In our study, ROC analysis of NT-proBNP showed an accuracy of 65% in predicting mortality (p - 0.016). Taking a cut-off of 10,000 pg/mL gave a sensitivity of 70% and a specificity of 52% in predicting mortality. While raised NT-proBNP appeared to predict mortality significantly on univariate analysis, multivariate logistic regression did not provide a significant correlation with one-month mortality. Noveanu et al. [18] found NT-proBNP at admission to be 72% accurate in predicting mortality at 30 days. Univariate analysis showed that NT-proBNP at 24 hours, 48 hours, and discharge significantly predicted mortality. However, on multivariate analysis, NT-proBNP levels at admission, 24 hours, or 48 hours did not significantly predict mortality, showing strong parallels with our study results. Peacock et al. [19] also concluded that testing for NT-proBNP did not reliably predict short-term mortality in ADHF, corroborating our study’s results. However, Luers et al. [20] concluded that serial change in NT-proBNP (increase in NT-proBNP levels within the first 12 hours of presentation) predicted 30-day mortality significantly as compared to a single NT-proBNP value at presentation. A systematic review by Santaguida et al. [21] also concluded that short-term mortality was not consistently predicted by NT-proBNP.

In our study, the mean EF in the group of patients who survived at one month was not significantly different from those who did not. An ejection fraction of less than 40% was not found to significantly predict mortality on logistic regression. Bhatia et al. studied 2,802 patients of AHF patients and found no significant difference in mortality rates between the HFpEF (HF with preserved EF, i.e., EF > 40%) and HFrEF (HF with reduced EF of less than 40%) groups at 30 days [22]. Similar findings were concluded by Owan et al. [23] and Cheng et al. [24], while conflicting results were concluded from very large registry data and meat analysis. Fonarow et al. [25] compared more than 20,000 patients of HFrEF with HFpEF from the OPTIMIZE-HF registry and demonstrated a significantly increased in-hospital mortality in the HFrEF group versus HFpEF group (3.9% versus 2.9%, p < 0.0001), but not the 60- and 90-day mortality. The Meta-analysis Global Group in Chronic Heart Failure (MAGGIC) published an individual patient data meta-analysis and found that the HFpEF patients had lower mortality than those with HFrEF (HR: 0.68, 95% CI: 0.64-0.71) [26]. Hence, the role of EF in predicting mortality in ADHF is not conclusive.

There are many novel biomarkers that are being postulated to be predictive of mortality in ADHF. Some of them include soluble suppression of tumorigenicity-2(sST2), galectin-3, copeptin, MR-proAdrenomedullin, and high sensitivity C-reactive protein. Studies show sT2 to be a significant, independent predictor of both cardiovascular and all-cause mortality in ADHF [27-29].

Our study had its share of limitations. A sample size of 100, while establishing a clear correlation between cTn-I and mortality in ADHF, was probably not enough to identify other probable predictors of mortality. Serial assessments of Troponin I and NT-proBNP were not done and their prognostic significance was not assessed in this study. Other suggested risk factors for all-cause in mortality include NYHA class IV presentation, lower systolic blood pressure, serum creatinine, and oxygen saturation at presentation [30]. As there was no blinding involved in the methodology of the study, observer bias could not be eliminated. While one-month mortality is indeed important to prognosticate, even more important from an emergency point of view would have been to identify in-hospital mortality or the mortality at a shorter interval. That would help in identifying further the patients that needed to be admitted or managed intensively at the ED level. Assessment of cardiovascular causes of mortality apart from all-cause mortality would have helped in better understanding the significance of biomarkers.

## Conclusions

In an emergency setting, Troponin I is a significant predictor of one-month mortality and serves better than NT-proBNP or left ventricular ejection fraction for the same, in patients with ADHF. cTnI values at presentation can hence be used to guide treatment, disposition, and follow-up decisions for these patients from the ED.
